# Computational verification of protein-protein interactions by orthologous co-expression

**DOI:** 10.1186/1471-2105-6-40

**Published:** 2005-03-02

**Authors:** Itay Tirosh, Naama Barkai

**Affiliations:** 1 Departments of Molecular Genetics and Physics of Complex Systems, Weizmann Institute of Science, Rehovot, Israel

## Abstract

**Background:**

High-throughput methods identify an overwhelming number of protein-protein interactions. However, the limited accuracy of these methods results in the false identification of many spurious interactions. Accordingly, the resulting interactions are regarded as hypothetical and computational methods are needed to increase their confidence. Several methods have recently been suggested for this purpose including co-expression as a confidence measure for interacting proteins, but their performance is still quite poor.

**Results:**

We introduce a novel computational method for verification of protein-protein interactions based on the co-expression of orthologs of interacting partners. The performance of our method is analysed using known *S. cerevisiae *interactions, and is shown to overcome limitations of previous methods. We present specific examples of known and putative interactions that are detected by our method and not by previous methods, and suggest that they represent transient interactions that might have been conserved and stabilized in other species.

**Conclusion:**

Co-expression of orthologous protein-pairs can be used to increase the confidence of hypothetical protein-protein interactions in *S. cerevisiae *as well as in other species. This approach may be especially useful for species with no available expression profiles and for transient interactions.

## Background

Protein-protein interactions (PPIs) have a central role in most biological processes, and identifying these interactions is an important goal of biological research. PPIs are the subject of extensive experimental studies, but the majority of them remain unknown. In the last few years, high-throughput techniques were developed for the identification of PPIs on a genomic scale. Yeast two-hybrid [1,2] and mass spectrometric analysis of protein complexes [3,4] were used to produce large sets of PPIs. However, these techniques are known to suffer from many false positives and the resulting PPIs are typically regarded as putative [5,6]. Thus, the development of computational methods for assessment and verification of putative PPIs is crucial [7-10]. Two such methods were proposed, that are based on the co-expression [11] and conservation [9] of PPIs, respectively. Here we propose to extend these methods by considering co-expression of orthologous protein pairs. We demonstrate the predictive power of our approach and discuss its advantages.

## Results

### Verification by mRNA co-expression

It was previously shown that interacting pairs of proteins are often correlated in their expression profiles [11,12]. The correlation of expression profiles was therefore proposed as a confidence measure for putative PPIs [7,10,13]. However, this approach has three major limitations. First, many pairs of non-interacting proteins are also co-expressed (false positives). Second, many pairs of interacting proteins are not co-expressed (false negatives). Third, to properly determine co-expression, mRNA expression profiles from a large and diverse set of conditions are needed, rendering this approach inapplicable for most organisms.

Former studies that used co-expression to identify PPIs did not explicitly examine its predictive power, or did not use a random set of protein-pairs as control for evaluating its performance. We thus carried out an analysis to evaluate the predictive power of this approach for *S. cerevisiae*, in order to later compare it to our new method. High quality *S. cerevisiae *expression data is available for many conditions, making it an ideal organism for the use of co-expression for validation of PPIs. We extracted a reference set of 1656 known interaction from the MIPS database [14], and generated a random set by randomly choosing pairs of proteins. Cosine correlation over our entire set of *S. cerevisiae *conditions was used to compare the levels of co-expression between the reference set and the random set (see methods).

**Figure 1:**
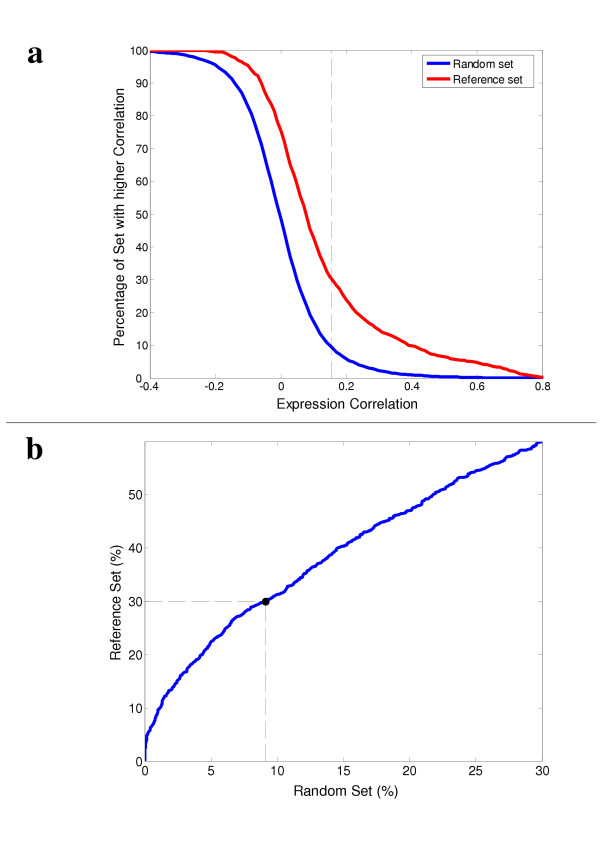
Higher correlation of expression profiles among interacting protein-pairs. (a) Cumulative distributions of correlations between expression profiles of protein-pairs from a reference set of 1656 known interactions taken from the MIPS database, and a set of randomly chosen pairs of proteins (averaged over ten trials). The dashed line represents a possible correlation threshold (0.155) that can be used for prediction of PPIs. (b) The predictive power of this approach. Each point in this plot represents a specific correlation threshold for the prediction of PPIs. The vertical axes shows the percentage of interaction identified from the reference set (true positives) and the horizontal axes shows the percentage of interaction identified from the random set (false positives). The dashed lines represent the performance of the threshold shown in (a).

The results of this analysis are summarized in Figure 1. The cumulative distributions of expression correlations in both sets are compared, showing higher degrees of co-expression in the reference set than in the random set (Figure 1a). The resulting predictive power is shown in Figure 1b, where each dot represents a possible correlation threshold for PPIs prediction. The percentages of protein-pairs passing each threshold from the random and reference sets are shown in the horizontal and vertical axes, respectively. For example, the threshold shown in Figure 1 (0.155) which leads to the correct verification of 30% of the reference set (497 true positives), results also in the false verification of approximately 9% of the random set (~149 false positives). Applying this to a set of putative PPIs with 50% false positives (as estimated for the *S. cerevisiae *yeast two hybrid sets [5,6]) results in a filtered subset with approximately 23% false positives (9% divided by 39%).

We verified that the performance of this method is largely independent of the exact set of conditions that is used, and that filtering the conditions or choosing them specifically for each pair of proteins does not improve the performance (not shown).

### Conservation of PPIs

Another approach that was proposed to verify or predict PPIs is based on conservation of interactions [9,15,16]. In this approach (termed "interologs"), pairs of proteins whose orthologs are known to interact in other species are assumed to interact. Such a method can potentially reveal many conserved PPIs, but it is currently limited by the availability and accuracy of interaction data. Without relying on putative interactions, the available set of *S. cerevisiae *PPIs only correspond to a small fraction of the biologically meaningful interactions, and the situation is much worse for other species. Consequently, this method has so far been based only on *S. cerevisia*e PPIs, including putative ones, to predict interactions in other organisms. Giot et al. used putative *S. cerevisia*e PPIs from mass spectrometric analysis to verify *Drosophila *PPIs found by yeast two-hybrid. Only 65 out of the ~2000 *Drosophila *putative interactions were identified as having an orthologous interaction in *S. cerevisia*e. This set was then used to train a statistical model for assignment of confidence scores to putative PPIs. Li et al. used putative *S. cerevisia*e PPIs gathered from several sources to predict *C. elegans *PPIs (rather than verify an existing set of putative PPIs). Out of the 5534 predicted *C. elegans *PPIs, only 949 were identified as having an orthologous interaction in *S. cerevisia*e [16].

The use of conserved interactions to verify a putative set of PPIs is therefore very limited, since only a small fraction of the putative set would have a known orthologous interaction. Furthermore, using putative PPIs in order to increase the coverage of this approach will decrease its accuracy and introduce many more false positives.

### Orthologous co-expression

#### Motivation

**Figure 2:**
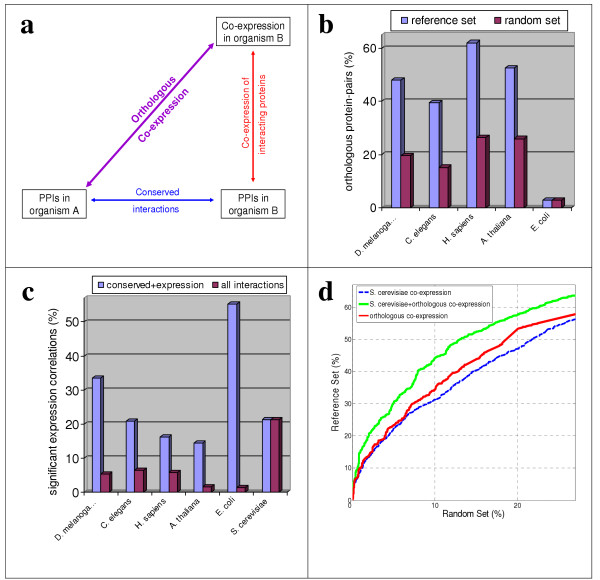
Orthologous co-expression can be used to predict PPIs. (a) Schematic representation of the method. (b) Interacting proteins are more likely to have an orthologous pair in other species. The percentage of yeast protein-pairs with an orthologous pair from five species (*C. elegans, E. coli, A. thaliana, D. melanogaster, and H. sapiens*) is shown for the reference and random sets. This property is seen for the four eukaryotes, but not for *E. coli*. (c) Orthologous pairs of interacting proteins are more likely to be co-expressed than orthologous pairs of random protein-pairs. The percentage of orthologous pairs having significant (P-value < 0.05) correlation of expression out of the total orthologous pairs with available expression data (conserved+expression), and out of the entire reference set (all interactions) is shown for all organisms (including S. cerevisiae). (d) Orthologous co-expression from five species was added and used to predict *S. cerevisiae *PPIs (red). The resulting predictive power is shown along with the predictive power of *S. cerevisiae *co-expression (dashed blue), as shown in Figure 1b. Orthologous co-expression was also added to *S. cerevisiae *co-expression, resulting in an improved predictive power (green).

In order to overcome the limitations of the two methods described above, we propose to integrate them and detect PPIs by orthologous co-expression, i.e. co-expression of the orthologs of the interacting partners (Figure 2a). A conserved interaction may be co-expressed only in a subset of the organisms in which it is present, so combining knowledge of co-expression from multiple organisms can be informative.

The use of orthologous co-expression for verification of PPIs is also supported by three previous observations. First, in order to preserve their interaction and functionality, interacting partners should co-evolve [17]. Sequence analysis was previously used to uncover co-evolution at the sequence level [18], but it may also be present at the level of gene expression. Second, as shown in two recent papers, co-expression of functionally linked proteins is more likely to be conserved than the co-expression of random pairs of proteins [19,20]. Hence, orthologous co-expression can replace co-expression, and serve as a better measure to identify functional links in general and PPIs in particular. Third, interacting protein-pairs are more likely to have pairs of orthologs in other species than randomly selected protein-pairs. This observation was made previously for different *ascomycota *species [21], and can also be seen in our analysis of more distant organisms (Figure 2b). Since orthologous co-expression can only be computed for conserved protein-pairs, the increased conservation of interacting protein-pairs will also increase the percentage of interacting pairs where orthologous co-expression can be computed, and lead to higher percentage of real PPIs out of the total predicted protein-pairs.

#### Performance

To examine whether orthologous co-expression can indeed be used to predict PPIs, we focused on *S. cerevisia*e orthologs from five species (*C. elegans, E. coli, A. thaliana, D. melanogaster*, and *H. sapiens*). Orthologous pairs of the protein-pairs in the reference and random sets were identified by BLAST [22], and their co-expression was measured using cosine correlation over the entire sets of mRNA expression data (see methods). Co-expression values of the random set orthologs in each organism were used to determine the 5% significance correlation thresholds. The percentage of interactions with significant orthologous co-expression in each organism (out of all the interactions where orthologous co-expression can be computed, i.e. interactions with both orthologs and expression profiles at that organism) is shown in figure 2c. Indeed, for all five organisms we found that orthologous-pairs of known PPIs are more likely to be co-expressed than that of random protein-pairs. Interestingly, the percentages of orthologous-pairs of PPIs with significant co-expression in *E. coli and D. melanogaster *are even higher than the percentage of PPIs with significant co-expression in *S. cerevisiae *(Figure 2c). Note, however, that less than 3% of the reference set had orthologous-pairs in *E. coli *and orthologous co-expression was computed only for 38 PPIs, so the high *E. coli *value might be a result of insufficient statistics.

The ability to predict PPIs by orthologous co-expression strongly depends on the percentage of interactions where orthologous co-expression can be computed (i.e. where both proteins are conserved and have expression profiles), so the percentages of PPIs that can be predicted by each organism is lower than 7% for all five organisms (Figure 2c). To overcome the lower coverage of each organism we combined the information from all five organisms. We examined the predictive power of this approach by repeating the analysis shown in Figure 1, when the yeast co-expression is replaced by the sum of the orthologous co-expression from the five other species (figure 2d). To avoid over-fitting, we only considered simple summation of the co-expression in different species. Notably, although *S. cerevisiae *co-expression was omitted from the analysis, the predictive power of this approach was better than that of *S. cerevisiae *co-expression alone (Figure 2d).

#### Combining *S. cerevisiae *and orthologous co-expression

The correlation between *S. cerevisiae *co-expression and orthologous co-expression of the true interactions in the test set is only 0.34. This means that the two methods are complementary, and that except for detecting interactions between co-expressed proteins, orthologous co-expression can also detect interactions between proteins that are not co-expressed in *S. cerevisiae*, but their corresponding orthologous are co-expressed in other species. Examples of known interactions from the test set with low co-expression in *S. cerevisiae *but high orthologous co-expression are shown in Table 1. In these 30 cases, the co-expression in *S. cerevisiae *is very low or even negative, but the orthologous co-expression is high in at least two species, such that they are easily detected by our approach.

Based on the complementarities of the two methods, namely *S. cerevisiae *and orthologous co-expression, we proceeded by adding the orthologous co-expression to *S. cerevisiae *co-expression (figure 2d). The addition significantly improved the results of both methods. Using the same example as mentioned above, the percentage of protein-pairs identified from the random set is reduced from 9% to 5%, while the percentage of proteins-pairs identified from the reference set remained 30%.

#### Transient interactions

In a previous study relating gene expression to PPIs, Jansen et al. classified protein complexes as 'permanent' and 'transient' [12]. The subunits of permanent complexes were shown to be highly co-expressed, in contrast to transient complexes where co-expression was very low. Transient interactions are therefore harder to detect by co-expression as well as by experimental methods.

**Figure 3:**
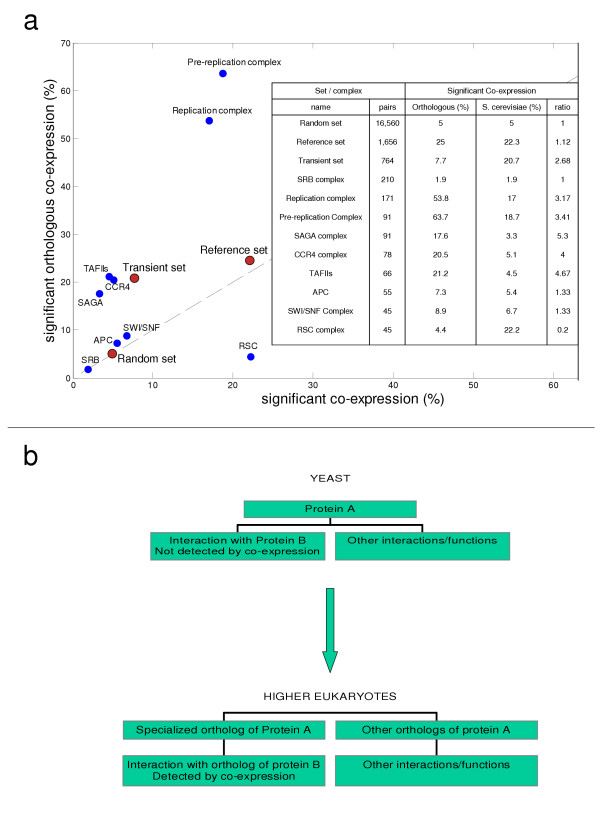
Detection of transient interactions. (a) Each circle shows the percentage of protein-pairs in a specific set/complex with a significant level (P-value < 0.05) of *S. cerevisiae *and orthologous co-expression in the horizontal and vertical axes, respectively. Blue circles represent all pair-wise interactions in a single transient complex; Red circles represent the three sets of protein-pairs (random, reference and transient). The dashed line indicates similar performance of both methods. The table also shows the number of protein-pairs in each set/complex, and the ratio between the percentage of pairs with significant orthologous and *S. cerevisiae *co-expression, respectively. (b) Proposed model for transient yeast interactions with low co-expression, but high orthologous co-expression. Protein A interacts with protein B, but also performs other functions or interacts with other proteins, such that it is not co-expressed with protein B. However, in higher eukaryotes, a specialized ortholog of A exist, which is co-expressed with the ortholog of B.

To test the performance of our method on transient interactions we examined the nine protein complexes classified as transient: pre-replication complex, replication complex, anaphase promoting complex (APC), TAFIIs, SAGA complex (Spt-Ada-Gcn5-acetyltransferase), CCR4 complex, RSC complex, SRB complex (kornberg's mediator) and SWI/SNF complex. Assuming all pair-wise interactions in these complexes, we compared the percentage of protein-pairs with significant *S. cerevisiae *or orthologous co-expression for each complex and for the combined set (Figure 3a).

Orthologous co-expression is slightly better than *S. cerevisiae *co-expression at identifying interactions in the reference set, but the differences in performance increase considerably when transient complexes are examined. In the combined set of 764 transient interactions, orthologous co-expression identifies almost three times (2.68) more interactions than *S. cerevisiae *co-expression. Moreover, for five out of the nine transient complexes, orthologous co-expression identifies at least three times more interactions than *S. cerevisiae *co-expression, while the opposite occurs only in one complex – RSC, which is also the smallest complex examined. These results suggest that orthologous co-expression is especially useful for detection of transient interactions.

#### Specialization of interacting proteins can lead to high orthologous co-expression

Why are there interacting protein-pairs which are not co-expressed in *S. cerevisiae*, while their corresponding orthologs are co-expressed in other species (Table 1; Figure 3a)? The observation that interacting protein-pairs are co-expressed is believed to be a result of their need to be present in similar amounts at different conditions. However, for transient interactions occurring only in specific processes, this requirement might affect only a small number of conditions, and hence might have a slight influence on the global levels of co-expression. In contrast, the orthologs of such interacting proteins might have adopted a stable interaction, resulting in co-expression at many conditions. Such transient interactions will not be detected by co-expression, and might also be hard to find using experimental methods, but orthologous co-expression may help to identify them. Moreover, one of the interacting proteins may be multifunctional, interacting with several proteins depending on context. The expression of such pleiotropic proteins is likely to be constitutive, and will not show correlation to that of its interacting partners. However, the pleiotropic protein might have several specialized orthologs in other species, each performing distinct functions, and co-expressed with the corresponding orthologs (Figure 3b). Note that in such cases the specialized ortholog may not be the closest one in sequence. However, allowing each protein to have multiple orthologs and choosing the maximal correlation can also increase the orthologous co-expression of false interactions. Consequently, such an approach only reduced the performance of our method (not shown).

#### Specific examples

To examine if specialization of interacting proteins can account for the high orthologous co-expression of protein pairs in Table 1 and in the transient complexes, we looked in more details at specific examples. Here we provide three examples supporting this notion.

**1. ***CDC28 *is the only cyclin-dependent kinase (CDK) in *S. cerevisiae *involved in cell cycle transitions [23]. *CDC28 *interacts with different proteins at different stages of the cell cycle, including G1 and B-type cyclins (*CLNs *and *CLBs*, respectively) and *CDC6*. Indeed, no detectable co-expression is found between *CDC28 *and its interacting partners (Table 1; not shown for *CLNs*). In contrast, *CDC28 *has several orthologs in higher eukaryotes (up to five distinct *CDKs *in mammals), each devoted to specific processes or tissues [23], and the orthologs that were found by our analysis in *H. sapiens*, *D. melanogaster *and *C. elegans (CDK2, CDC2 *and *CDK-1*, respectively) are highly co-expressed with the corresponding orthologs of *CDC6 *and the B-type cyclins (Table 1).

**2. **Yeast *TAF5 *is a component of at least two transient complexes, the general transcription factor TFIID and the SAGA complex [24]. However, its human ortholog (*TAF5*) is only known to be a part of the TFIID complex, while a second ortholog (*TAF5L*) is known to be in both TFIID, and the human equivalent of SAGA [25]. As expected, the co-expression of human *TAF5 *and the other proteins in human TFIID is higher than that of yeast *TAF5 *and the other proteins in yeast TFIID (not shown).

**3. **The opposite case of two *S. cerevisiae *paralogs with only one ortholog in higher eukaryotes, though less common, may also help to identify PPIs. The nascent polypeptide associated complex (NAC), consists of an alpha subunit (*EGD2*) and a beta subunit (either *EGD1 *or *BTT1*) [26]. *BTT1 *is not co-expressed with *EGD2*, presumably since *EGD1 *and *BTT1 *are alternating beta subunits that bind both the ribosome and the alpha subunit (*EGD2*). In contrast, *D. melanogaster *and *C. elegans *have only one known orthologous beta subunit, which are co-expressed with the corresponding orthologs of *EGD2 *(Table 1).

#### Predictions

Table 2 shows examples of low confidence putative interactions with low co-expression but high orthologous co-expression. These interactions were found by high-throughput yeast two-hybrid [1], and considered low confidence (they had less than 3 interaction sequence tags and were not included in the core data; also not supported by co-expression). However, in light of the high orthologous co-expression from at least two species, we predict that they represent true interactions. In support of that, both proteins in all these examples are localized to the same cellular compartment (according to the MIPS database [14]).

Some of these proposed interactions might also fit the model in Figure 3. For example, *SMT3 *is the only SUMO gene in *S. cerevisiae*, which is known to modify *TOP2 *(DNA Topoisomerase II) and other proteins [27]. However, in vertebrates there are three known SUMO genes: *SUMO1*, *SUMO2*, and *SUMO3*. As suggested by the model in Figure 3, *SMT3 *is not co-expressed with *TOP2*, but one of its human orthologs (*SUMO1*), is highly co-expressed with the human ortholog of *TOP2 *(*TOP2A*; see Table 2).

## Discussion

We presented here a new computational method for verification of PPIs that is based on the co-expression of orthologous protein-pairs, and demonstrated its predictive power using PPIs identified in *S. cerevisiae*.

This method extends two of the former methods, namely co-expression of interacting proteins and conservation of interactions (interologs). The first method can only be applied to organisms with expression data and its performance depends on the amount and quality of that data. Our method overcomes this limitation by integrating sequence and expression data from other organisms. It can thus be applied to any sequenced organism, particularly for those without available expression data, thereby replacing the missing data. Moreover, it performs better than the former method even for *S. cerevisiae*, where many high quality expression data is available, and is especially better in identifying transient interactions. It is difficult to evaluate our approach for other species, since we do not have large representative sets of known interactions, but the success in yeast is promising.

The proposed method also overcomes the limitation of the interologs approach, namely the small fraction of interactions that is known to date. Our method uses expression rather than interaction data, which makes it capable of giving evidence for a much larger number of interactions.

mRNA expression profiles are being generated by many different labs for a wide range of organisms. The improved quality of existing expression profiles as well as the addition of profiles for other organisms will improve the performance of our method. Further improvements can be achieved by giving different weights to the co-expression from different organisms (not shown). A weight can be given to each organism according to the reliability of its expression profiles, or according to its evolutionary distance from the studied organism.

During the writing of this manuscript, a related approach was suggested [28]. Based on the codon adaptation index (CAI) as an estimator for average expression levels, Fraser et al. examined co-evolution of expression levels from four fungi closely related to *S. cerevisiae*, and used that to predict PPIs in *S. cerevisiae*. This approach is complementary to the one that we have proposed. Thus, mRNA expression should be used directly when possible, even from relatively distant species (such as *D. melanogaster*), and CAI should be used from closely related species without available expression data.

Finally, the methods described here are still not accurate enough to verify specific PPIs, but they provide additional evidences and are useful for assessment and filtering of high-throughput PPIs data sets, in order to produce smaller sets of higher confidence, and direct further investigations. Complementary methods should be combined to create a general scheme for verification of putative PPIs, for example by considering only those interactions that are verified by at least two or three methods [7] or using supervised machine learning approaches [29], thus improving the performance of each method alone.

## Conclusion

We have shown that expression data from multiple organisms can be used to increase the confidence of hypothetical PPIs by considering co-expression of orthologs of the presumed interacting partners. For organisms such as *S. cerevisiae*, with highly characterized expression profiles, orthologous co-expression may be combined with co-expression of the actual proteins, whereas for other, less studied organisms, it may replace the missing expression profiles. Notably, this method is especially useful for detection of transient interactions which presents a known weakness of most prediction methods. The success of this method also implies that PPIs tend to be conserved in different organisms, even as distant as yeast and human, further supporting the use of comparative approaches in proteomics.

## Methods

*Interactions sets *– a reference set of *S. cerevisiae *interactions was extracted from the MIPS (Munich Information Center for Protein Sequences) PPI database [14] at 22/01/04. We excluded genetic interactions, self-interaction, interactions found by high-throughput experiments, interactions without expression data, and redundancies, resulting in a set of 1656 interactions. We did not use larger databases such as the one compiled by von Mering et al. [7] since they are more likely to contain false interactions and are also biased towards co-expression since this information was used in their construction. Randomly generated set of the same size was used as control, and averaged over ten trials. Self-interactions were excluded from the random set. The random set may include real interaction, but their expected frequency is much less than 1%. Transient complexes were taken from Jansen et al. [12]. The transient set was constructed by combining the pair-wise interactions from each transient complexes and removing redundancies (some protein pairs were present in more than one complex).

*mRNA expression data *– datasets for six organisms were collected from different sources, as described in [19], and can be downloaded from our home page [30]. All datasets were normalized to have a mean of 0 and standard deviation of 1 for each condition.

*Expression correlation *– cosine correlation over the entire expression data of each organism was used as a measure of co-expression. Former analysis suggested that cosine correlation is the optimal measure of co-expression for the purpose of detecting PPIs [13]. Many genes in all six organisms have missing values in the expression data, so the expression correlations of many orthologous pairs cannot be calculated. To decrease the dependency of our approach in the availability of expression data and to improve its performance, we replace the missing correlations by estimated values. We used the corresponding yeast co-expression when the yeast and orthologous co-expression are combined (green curve in figure 2d). In contrast, when orthologous co-expression is used alone (red curve in figure 2d), the yeast expression data is assumed to be unavailable (in order to show the applicability of the method to organisms without expression data) and an expected correlation is calculated for each species, based on the union of the reference and random sets (average expression correlation of orthologous pairs in a specific species, over the reference and random sets combined with equal weights). The expected correlations are greater than zero for all five species; so putative PPIs are actually given positive scores for the existence of an orthologous pair, corresponding to the notion that PPIs are more likely to have pairs of orthologs [21].

*Orthologous proteins *– orthologs were found using blastp [22] with a P-value threshold of 10^-7^, and alignment length threshold of 0.3. The ortholog with the most significant p-value that had available expression data was used to measure co-expression. Other studies had used a reciprocal best-hit BLAST search for finding orthologous; we use a less strict criterion in order to apply the orthologous co-expression method to more protein-pairs.

*P-values and Significance *– by sampling 100,000 protein pairs we determined p-values for *S. cerevisiae *and orthologous co-expression as the fraction of pairs with equal or greater correlation of expression profiles; P-values of 0.05 (not corrected for multiple testing) were used as thresholds for significance.

## Figures and Tables

**Table 1:** *S. cerevisiae *and orthologous co-expression of known Protein interactions

**GENE 1**	**GENE 2**	***S. cerevisiae *Co-expression**		**Orthologous Co-expression**			
		
		**correlation**	**p-value**	***D. melanogaster***	***C. elegans***	***H. sapiens***	**p-value**
*CDC28*	*CLB2*	-0.07	0.74	*	0.77	0.59	3.7e-04
	*CLB4*	-0.06	0.71	0.85	0.77	0.59	1.0e-05
	*CDC6*	-0.03	0.60	0.77	0.32	0.37	2.6e-04
	*CLB3*	-0.01	0.52	0.85	0.77	0.59	1.0e-05
	*CLB1*	0.05	0.30	*	0.65	0.59	7.3e-04
	*CLB5*	0.06	0.27	0.87	0.65	0.45	3. 0e-05

*DMC1*	*PDC5*	-0.09	0.80	0.59	*	0.17	6.8e-03
	*PDC1*	-0.05	0.67	0.59	*	0.17	6.8e-03
	*RIS1*	-0.02	0.56	*	0.34	0.52	4.3e-03

*PRP9*	*RIS1*	0.02	0.41	*	0.28	0.62	3.7e-03
	*PRP11*	0.05	0.30	0.88	0.35	0.33	1.6e-04
	*NOG2*	0.07	0.24	0.41	0.33	0.66	3.5e-04

*SSN6*	*CUS1*	-0.06	0.71	0.76	0.32	*	5.4e-04
	*SNP1*	0.08	0.21	0.79	0.21	*	2.1e-03

*PAB1*	*SGN1*	-0.17	0.93	0.56	0.19	0.26	2.4e-03
	*RNA14*	-0.06	0.71	0.42	0.17	0.24	5.8e-03
*PFS2*	*RNA14*	-0.03	0.60	0.44	0.31	-	8.5e-03
*HAT1*	*HAT2*	-0.04	0.33	0.86	0.19	0.22	7.3e-04
*SIT4*	*TAP42*	-0.16	0.92	0.54	-	0.36	4.3e-03
*TRS23*	*BET3*	-0.10	0.82	*	0.20	0.52	8.1e-03
*DNA2*	*RAD27*	-0.01	0.52	*	0.41	0.47	3.9e-03
*PRP8*	*SNU114*	0.05	0.30	*	0.60	0.57	8.7e-04
*HRB1*	*MTR10*	-0.07	0.74	0.56	0.27	0.27	1.5e-03
*BTT1*	*EGD2*	-0.08	0.77	0.42	0.52	0.59	2.0e-04
*GPA1*	*STE11*	0.07	0.24	0.55	0.25	0.37	1.1e-03
*UBA2*	*AOS1*	0.08	0.21	0.74	0.17	0.41	5.4e-04
*SPT15*	*BRF1*	0.09	0.19	0.72	0.21	0.23	1.1e-03
*TAF5*	*TAF9*	0.05	0.30	0.86	-	0.18	2.1e-03
*LSM5*	*KEM1*	0.04	0.33	-	0.37	0.65	2.3e-03
*RPB3*	*MED7*	0.01	0.45	*	0.49	0.32	5.3e-3

* denotes that at least one of the corresponding orthologs did not have expression data.

- denotes that there is no pair of corresponding orthologs.

**Table 2:** *S. cerevisiae *and orthologous co-expression of hypothetical Protein interactions

**GENE 1**	**GENE 2**	***S. cerevisiae *Co-expression**		**Orthologous Co-expression**			
		
		**correlation**	**p-value**	***D. melanogaster***	***C. elegans***	***H. sapiens***	**p-value**
*TAF5*	*PIF1*	-0.05	0.67	0.74	0.05	0.42	8.7e-04
*COR1*	*XDJ1*	-0.01	0.52	*	0.43	0.39	5.0e-03
*PUS2*	*LPD1*	-0.06	0.71	*	0.23	0.55	6.1e-03
*TAF6*	*PUB1*	0.00	0.49	0.04	0.41	0.50	3.3e-03
*PAN3*	*YNL092W*	-0.04	0.64	0.77	0.25	*	1.9e-03
*SMT3*	*TOP2*	-0.08	0.77	*	0.47	0.69	9.6e-04

* denotes that at least one of the corresponding orthologs did not have expression data.
